# Systemic antibiotics for diabetes mellitus‐related foot infections: an integrative literature review

**DOI:** 10.1590/1677-5449.202500972

**Published:** 2026-03-23

**Authors:** Catarina França dos Santos, Vanessa Prado dos Santos, Lucas de Mello Ferreira, Neima Prado dos Santos, André Brito Queiroz, Valter Castelli, Roberto Augusto Caffaro, Carlos Alberto Silveira Alves

**Affiliations:** 1 Universidade Federal da Bahia – UFBA, Salvador, BA, Brasil.; 2 Irmandade Santa Casa de Misericórdia de São Paulo – ISCMSP, São Paulo, SP, Brasil.

**Keywords:** antibiotics, diabetic foot, infection, treatment, diabetes mellitus

## Abstract

Evidence on antibiotic therapy for diabetic foot infections can help clinical management. The objective of this study was to identify the evidence on systemic antibiotics for treatment of diabetes mellitus-related foot infections. An integrative literature review was conducted of randomized clinical trials, systematic reviews, and meta-analyses. The keywords “Diabetic foot” AND “Antibiotics” were used to search PubMed and 15 articles were selected (nine randomized clinical trials, four systematic reviews, and two meta-analyses). Seven randomized clinical trials revealed clinical results that were comparable for beta lactam antibiotics with beta-lactamase inhibitors, carbapenems, and fluoroquinolones. Two randomized clinical trials found significant differences comparing ertapenem and tigecycline and in analyses of subsets with severe infections between piperacillin-tazobactam and ertapenem. The literature revealed comparable clinical results for different systemic antibiotics used to treat foot infections related to diabetes, except for the difference between ertapenem and tigecycline, which did not meet the parameters for non-inferiority, highlighting the need for higher-quality evidence.

## INTRODUCTION

Diabetes mellitus (DM) constitutes a worldwide public health challenge with growing prevalence. In 2021, there were 529 million people with DM and it is estimated that this number will exceed 1.31 billion in 2025.^1^ The age-standardized prevalence of DM increased 90.5% from 1990 to 2021, rising from 3.2% to 6.1%; in South America this increase was greater than 100%.^1^ The majority of cases (96.0%) are type 2 DM and, in general, prevalence is higher among men, except in some geographic regions, such as Central Latin America , South Sub-Saharan Africa, and the Caribbean, where prevalence is higher among women.^1^

Hyperglycemia in DM is linked to a range of acute and chronic complications, including diabetic foot ulcers. The estimated prevalence of DM-related foot ulceration is 6.3% and is higher among people with type 2 DM than those with type 1 (6.4% vs. 5.5%).^2^ Diabetic foot ulcers can be caused by different etiologies, including peripheral neuropathy, ischemia due to vascular disease, or a combination of both factors, resulting in neuroischemic ulcers, which are the most common type.^3^ It is estimated that 19 to 34% of people with DM will develop a foot ulcer or wound over the course of their lives.^3,4^

There are many classifications of diabetic foot ulcers in the literature, based on a variety of factors that denote their severity, such as size and depth of ulcer, neuropathy, ischemia, and presence of infection.^3^ Ischemia and infection make treatment more complex and increases the risk of amputations.^3,5^ Infections of diabetic foot ulcers can be classified as mild, moderate, or severe, according to the local and systemic characteristics of the disease.^6^ For infected ulcers, in addition to wound care, it is often necessary to introduce empirical antibiotic therapy until the results of cultures and antibiotic resistance tests are available.

For moderate and severe infections, the International Working Group on the Diabetic Foot (IWGDF) guidelines recommend cultures, preferably using tissue specimens, to guide antibiotic therapy targeting the pathogen responsible for the infection.^7^ Selection of antibiotic therapy for diabetic foot ulcers should take several factors into consideration, such as clinical parameters of the patient, the severity of the wound, prior use of antibiotics, and the microorganisms most likely to be involved, in addition to knowledge of literature on the efficacy of different antibiotics agents for this type of infection.^7,8^

The objective of this study was to conduct an integrative literature review to identify what scientific evidence exists on the efficacy of systemic antibiotic treatment for diabetic foot infections. The intention is to determine whether the evidence in the literature supports selection of specific antibiotics for treatment of infected foot ulcers in patients with DM.

## METHODS

The study employed integrative literature review methodology using the criteria established in the Preferred Reporting Items for Systematic Reviews and Meta-Analyses for the systematic reference searches.^9^

### Search strategy

The Medical Subject Headings (MeSH) keywords “Diabetic foot” AND “Antibiotics” were chosen. The PubMed database was used to conduct the search for references in three steps, each using the same keywords. The first step comprised a search with the clinical trials and randomized clinical trials (RCTs) filter applied, the second step used the systematic reviews (SRs) filter, and the third step used the meta-analyses filter. The last of these steps was completed on January 8, 2025.

### Eligibility criteria

Clinical trials (randomized or otherwise), SRs, and meta-analyses were considered eligible if they described comparisons between different systemic antibiotics used to treat infected diabetic foot ulcers. Articles were not considered eligible if they compared topical antibiotics, studied the duration of antibiotic therapy, or analyzed combinations with other types of treatment for foot ulcers in people with DM.

### Article selection

An initial search of the PubMed database using the keywords, but without the filters chosen for the three stages, returned 2,200 results. Applying the filters reduced this number to 212 items and application of the selection criteria identified 15 publications, which were selected for the review. All article titles were read to determine eligibility based on the study objective and selection criteria and then the abstracts of those deemed eligible were read considering the same criteria.

**Figure 1:**
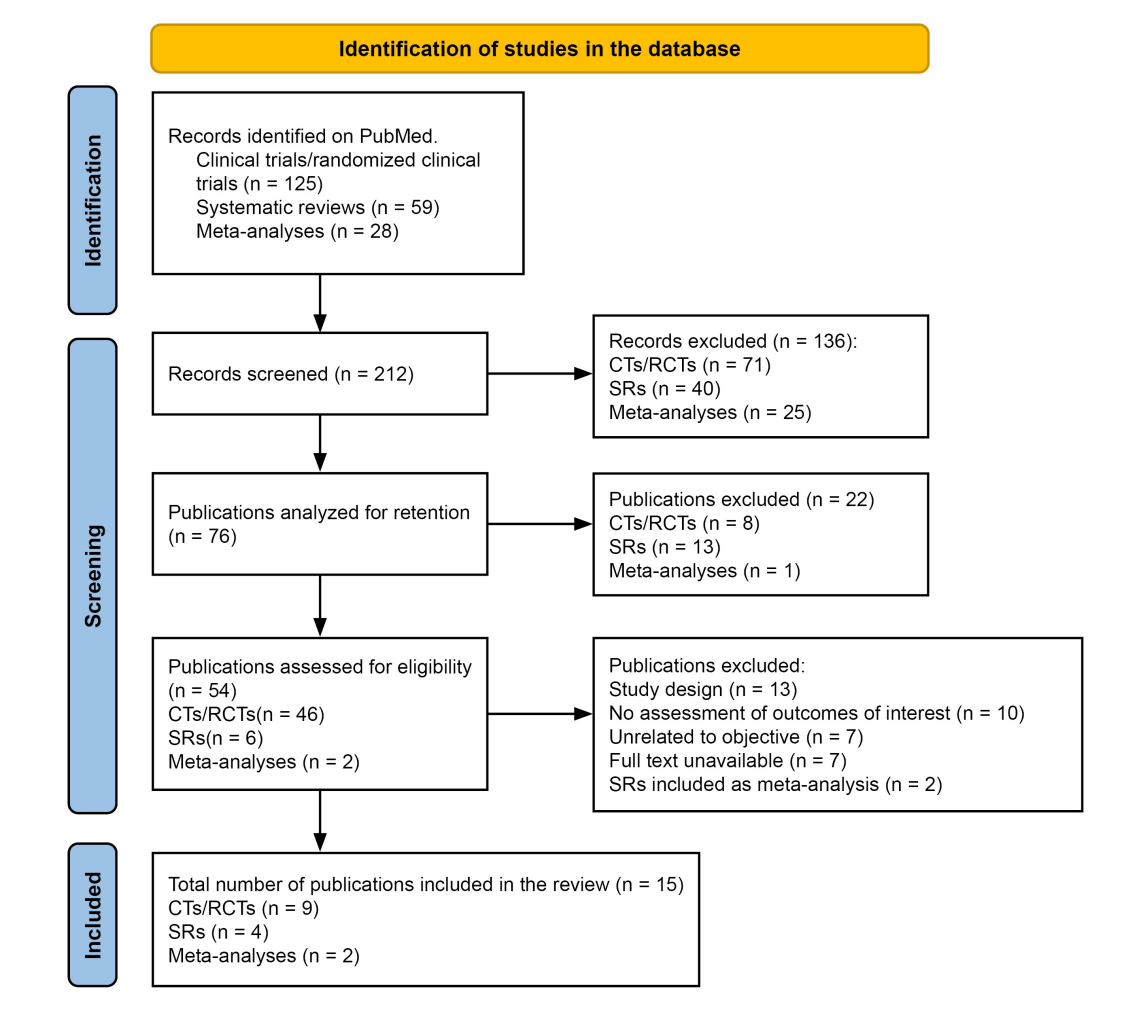
Flow diagram illustrating the process of selection of the articles included in the integrative literature review.^11^ CT = clinical trial; RCT = randomized clinical trial; SR = systematic review.

After all of the search stages and application of the eligibility criteria, nine RCTs, four systematic literature reviews, and two meta-analyses were selected for analysis in the integrative review. One of the included SRs^10^ contained a literature review conducted to update the International Working Group on the Diabetic Foot (IWGDF) guidelines, developed in conjunction with the Infectious Diseases Society of America.^7^Figure 1 shows a flow diagram detailing the process for selection of publications. The article search and selection procedures were conducted by two researchers independently. Any disagreements were resolved by discussion among the authors. A Microsoft Excel spreadsheet was used for extraction and analysis of the data from the studies.

### Methodology for analysis of the selected articles

The 15 selected articles were analyzed with the aid of the Microsoft Excel spreadsheet used for data extraction, which was populated with detailed information on the studies included, covering their principal characteristics and the main findings of the comparisons of different systemic antibiotics. Information was extracted on authors, year of publication, journal, sample size, objectives, methodology, systemic antibiotics compared, efficacy of antibiotics/clinical success, and authors’ conclusions. Approximate clinical success percentages were calculated mathematically for use in graphs. The articles included were analyzed descriptively and their data were displayed using Tableau® software.

## RESULTS

### Randomized clinical trials

**Figure 2:**
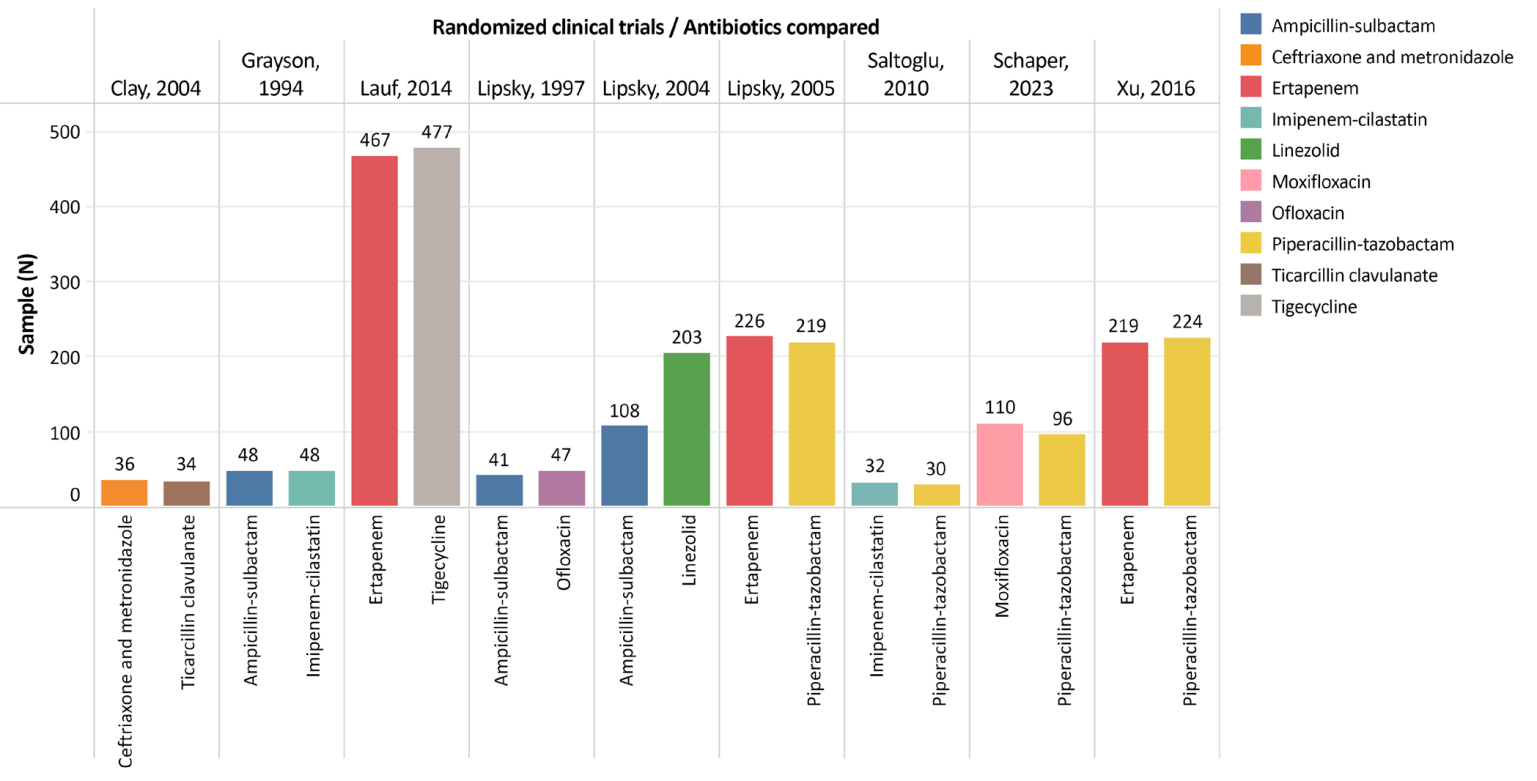
Graph illustrating the sample size for each antibiotic compared in the nine randomized clinical trials.

A total of nine RCTs compared the efficacy of different systemic antibiotics for treatment of infected foot wounds in people with DM (Table 1).^12-20^ The sample sizes analyzed in these RCTs varied from 62 to 944 cases (Figure 2). The method used to classify the ulcers treated with antibiotic therapy differed between the nine RCTs (Table 2). Two RCTs mentioned the Wagner scale,^12,18^ three described ulcers as the most common type of lesions,^13,15,16^ two only reported the University of Texas classification,^17,20^ one reported the PEDIS classification (perfusion, extent/size, depth/tissue loss, infection, and sensation),^14^ and one used two classifications (PEDIS and University of Texas ).^19^

**Table 1:** Synthesis of the objectives, systemic antibiotics compared, and conclusions of the included randomized clinical trials, systematic reviews, and meta-analyses.

**Authors**	**Summary of objectives**	**Sample analyzed/number of studies included**	**Antibiotics compared**	**Summary of conclusions**
**Randomized clinical trials**				
Clay et al.^12^	To compare metronidazole + ceftriaxone vs. ticarcillin/clavulanate	70	Metronidazole + ceftriaxone vs. ticarcillin/clavulanate	Metronidazole + ceftriaxone was as effective as ticarcillin/clavulanate
Grayson et al.^13^	To compare imipenem/cilastatin vs. ampicillin/sulbactam	96^a^	Imipenem/cilastatin vs. ampicillin/sulbactam	Ampicillin/sulbactam and imipenem/cilastatin had similar efficacy
Lauf et al.^14^	To compare tigecycline vs. ertapenem (with or without vancomycin)	944	Tigecycline vs. ertapenem (with or without vancomycin)	Tigecycline did not meet the criteria for non-inferiority to ertapenem (± vancomycin)
Lipsky et al.^15^	To compare floxacin (followed by oral ofloxacin) vs. ampicillin/sulbactam (followed by oral amoxycillin clavulanate)	88	Ofloxacin (followed by oral ofloxacin) vs. ampicillin/sulbactam (followed by oral clavulanate/amoxycillin)	Both regimes were effective and led to cure in the majority of cases
Lipsky et al.^16^	To compare linezolid vs. aminopenicillin/betalacmatase inhibitor	311	Linezolid vs. ampicillin/sulbactam and/or amoxycillin/clavulanate (could be combined with vancomycin or aztreonam)	Linezolid was as effective as aminopenicillin/beta-lactamase inhibitors
Lipsky et al.^17^	To compare ertapenem vs. piperacillin/tazobactam	445	Ertapenem vs. piperacillin/tazobactam	Ertapenem was as effective as piperacillin/tazobactam
Saltoglu et al.^18^	To compare piperacillin/tazobactam vs. imipenem/cilastatin	62	Piperacillin/tazobactam vs. imipenem/cilastatin	Although there was better clinical response to piperacillin/tazobactam, the difference was not statistically significant
Schaper et al.^19^	To compare moxifloxacin vs. beta lactamic antibiotic/beta-lactamase inhibitor	206	Moxifloxacin (followed by moxifloxacin oral) vs. piperacillin/tazobactam (followed by amoxycillin/clavulanate)	Moxifloxacin was effective and similar to piperacillin/tazobactam.
Xu et al.^20^	To compare ertapenem vs. piperacillin/tazobactam	443	Ertapenem vs. piperacillin/tazobactam	Ertapenem was not inferior to piperacillin/tazobactam in general, but had a significantly smaller clinical resolution rate for severe infections
**Systematic reviews**				
Nelson et al.^21^	Clinical- and cost-effectiveness of antibiotic agents for diabetic foot ulcers	23	Imipenem-cilastatin, piperacillin-tazobactam, cefazolin, ceftriaxone, ampicillin-sulbactam, cefoxitin, linezolid, ticarcillin-clavulanate	The evidence is too weak to recommend a specific antibiotic agent.
Tchero et al.^22^	Efficacy of topical and systemic antibiotics for diabetic foot infections	16	Piperacillin-tazobactam (followed or not by amoxycillin-clavulanate), moxifloxacin, ertapenem, ampicillin-sulbactam, imipenem-cilastatin, ticarcillin-clavulanate, ceftriaxone (with metronidazole), tigecycline	Ertapenem had better results than tigecycline, but was inferior to piperacillin/tazobactam for treatment of severe infections
Pratama et al.^23^	Efficacy of different antibiotic regimes in patients with infected diabetic foot ulcers	16	Ertapenem, piperacillin-tazobactam, ampicillin-sulbactam, moxifloxacin, amoxycillin-clavulanate, imipenem-cilastatin, tigecycline, ertapenem, ceftriaxone, levofloxacin	There is no strong evidence to recommend a specific antibiotic with greater efficacy
Peters et al.^10^	Therapeutic interventions for the IWGDF guidelines^b^ on diagnosis and treatment of diabetic foot infections	32^c^	Beta lactam antibiotics/beta-lactamase inhibitors; imipenem-cilastatin; cefoxitin; ofloxacin; linezolid; ertapenem; tigecycline; moxifloxacin	There was no difference in the results of the antibiotics compared, except in the study that found tigecycline was inferior to ertapenem (with or without vancomycin).
**Meta-analyses**				
fGurusamy et al.^24^	To compare antibiotics for treatment of non-surgical wounds with infection or colonization by MRSA^d^	3	Daptomycin, vancomycin, semi-synthetic penicillin, ertapenem, moxifloxacin, piperacillin-tazobactam followed or not by amoxycillin-clavulanate	There is no evidence to recommend a specific antibiotic for infections of non-surgical MRSA infections.^c^
Selva Olid et al.^25^	To compare systemic antibiotics for treatment of diabetic foot infections against other systemic antibiotics, topical antibiotics, or placebo	20^e^	Piperacillin-tazobactam; ticarcillin-clavulanate; ampicillin-sulbactam; aztreonam; imipenem-cilastatin; cefoxitin; ceftobiprole; ceftazidime; vancomycin; ceftriaxone + metronidazole; ertapenem; moxifloxacin; cinafloxacin; amoxycillin-clavulanate; levofloxacin; ofloxacin; daptomycin; linezolid; clindamycin; cephalexin; tigecycline	No specific antibiotic was identified that had better results for resolution of infections; one study showed that ertapenem (± vancomycin) was more effective than tigecycline

MRSA = Methicillin-resistant *Staphylococcus aureus*; IWGDF = International Working Group on the Diabetic Foot.

a 96 events/92 patients.

b IWGDF.

c 26 studies in the analysis for the review on beta lactam antibiotics.

d MRSA.

e 20 studies/24 articles.

f According to the authors, it was not possible to conduct a meta-analysis.

**Table 2:** Antibiotics, wound classifications, and main microbiological results from the randomized clinical trials included in this integrative review.

**Authors**	**Clinical success of the antibiotics compared (%)**	**Sample analyzed**	**Lower limb wound classifications**	**Predominant pathogen type**	**Main species/genera of microorganisms isolated (n)**
**Randomized clinical trials**					
Clay et al.^12^	Ceftriaxone + metronidazole (72%) vs. ticarcillin-clavulanate (76%)	70	Wagner 1-3 (71% of the patients had cellulitis of the foot)	Gram-positive	*Staphylococcus aureus* (20); Group B *streptococcus* (9); *Enterococcus faecalis* (6)
Grayson et al.^13^	Imipenem-cilastatin (85%) vs. ampicillin-sulbactam (81%)	96^a^	Ulcers (88 cases)	Gram-positive	*Staphylococcus aureus* (54); *Enterococcci* (35); *Streptococci* (28)
Lauf et al.^14^	Tigecycline (77.5%) vs. ertapenem (82.5%) (with or without vancomycin)	944	PEDIS infection grades 2-4 (90% PEDIS 2 or 3)	Gram-positive	*Staphylococcus aureus* (323); *Enterococcus faecalis* (134); *S. agalactiae* (88)
Lipsky et al.^15^	Ofloxacin (85%) (followed by oral ofloxacin) vs. ampicillin-sulbactam (83%) (followed by oral amoxacillin-clavulanate)	88	Infected ulcers (48 cases), cellulitis (26 cases)	Gram-positive	*Staphylococcus aureus* (41); *Streptococcus sp*. (24); *Coagulase-negative staphylococcus* (16)
Lipsky et al.^16^	Linezolid (81%) vs. aminopenicillin/beta-lactamase inhibitors (71%)	311	Infected ulcers (283 cases), cellulitis (161 cases)	Gram-positive	*Staphylococcus aureus* (131); *Coagulase-negative staphylococcus* (54); *Enterococcus sp.* (51)
Lipsky et al.^17^	Ertapenem (94%) vs. piperacillin-tazobactam (92%)	445	University of Texas (most common, moderate grade I, stage-B: 354 cases)	Gram-positive	*Staphylococcus aureus* (147), *Peptostreptococcus magnus* (65), *Streptococcus agalactiae* (46)
Saltoglu et al.^18^	Piperacillin-tazobactam (46,7%) vs. imipenem-cilastatin (28.1%)	62	Wagner 2-4 (34 cases class 3; 19 cases class 4)	Gram-negative	*Coagulase-negative staphylococcus* (15); *Pseudomonas aeruginosa* (13); *Enterococcus sp*. (10)
Schaper et al.^19^	Moxifloxacin (76.4%) vs. piperacillin-tazobactam (78.1%)	206	PEDIS grades 2-4; University of Texas (Grade II-ischemic: 88 cases and Grade II-infected: 30 cases)	Gram-positive	*Staphylococcus aureus* (133), *Enterococcus faecalis* (59), *Escherichia coli* (21)
Xu et al.^20^	Ertapenem (93.6%) vs. piperacillin-tazobactam (97.3%)	443	University of Texas (Severe-grade 2/ stage B:131 cases; Moderate-grade 1/stage B: 106 cases)	Gram-positive	*Staphylococcus aureus* (126); *Enterococcus faecalis* (72); *Escherichia coli* (51)

a 96 events/92 patients.

PEDIS = perfusion, extent/size, depth/tissue loss, infection and sensation.

**Figure 3:**
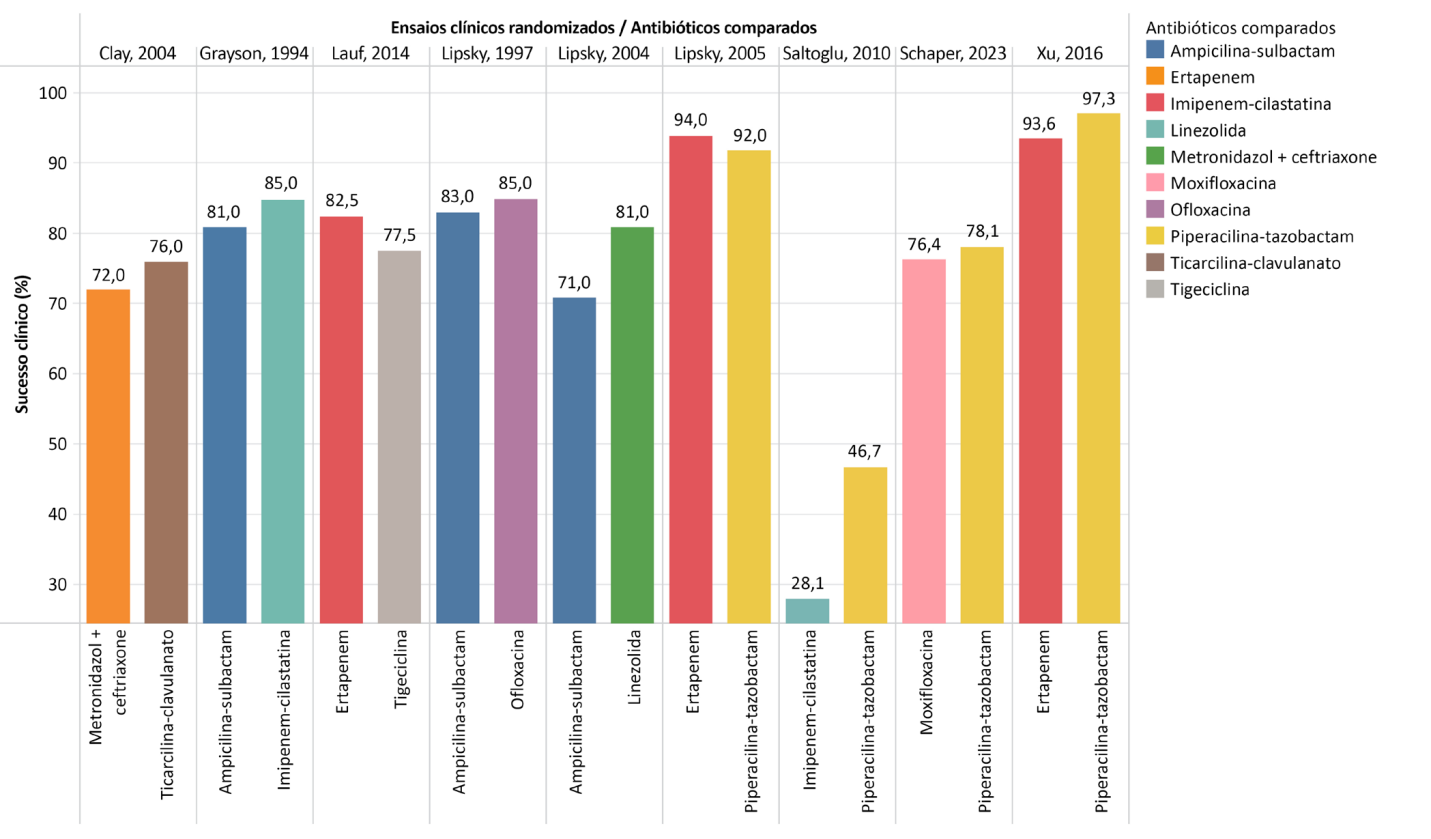
Graph illustrating treatment results (clinical success percentages) for the antibiotics compared in the nine randomized clinical trials.

The RCTs differed in the way they assessed the results of antibiotic therapy, describing symptom improvement, clinical cure, or favorable clinical response, using different criteria to define outcomes. The classes of antibiotics most often compared were beta lactam antibiotics combined with betalactamase inhibitors (eight studies), followed by carbapenems (five studies), fluoroquinolones (two studies), and cephalosporins, oxazolidones, and glycylcyclines (one study each). In the RCT comparing linezolid vs. aminopenicillin with a beta-lactamase inhibitor, vancomycin or aztreonam could be added, at the team’s discretion.^16^ The rates of efficacy, clinical success, or favorable result in the nine RCTs ranged from 28.1 to 97.3% for the different antibiotics compared (Table 2 and Figure 3).

In general, eight of the nine studies did not report significant differences between the clinical success achieved with the different antibiotics compared. The RCT that did observe a significant difference found that tigecycline did not meet the criteria for non-inferiority when compared to ertapenem, which could be combined with vancomycin.^14^ In the group given tigecycline (408 cases), 316 (77.5%) were considered cured, compared to 334 (82.5%) of the 405 cases treated with ertapenem ± vancomycin.^14^ Vancomycin was administered as an adjuvant to ertapenem in 15.6% of cases.^14^ This was the RCT^14^ with the largest number of patients and had the largest sample of the included studies (Figure 2).

The comparison of ertapenem vs. piperacillin-tazobactam in the RCT^20^ that recruited 443 patients with moderate to severe infections did not detect any significant difference in clinical response for the whole sample (93.6% vs. 97.3%), but did find a significantly lower rate of favorable response to ertapenem in an analysis of a subset (273 cases) with severe infections (91.5% vs. 97.2%; p = 0.04).^20^ With regard to the microbiological aspects of the ulcers, Gram-positive microorganisms predominated in eight RCTs, particularly *Staphylococcus aureus* (Table 2). In the only RCT^18^ in which Gram-negative bacteria predominated, the clinical results revealed lower success rates than in the other studies (Figure 3).

### Systematic reviews

The four SRs included in this analysis included studies using systemic and topical antibiotics.^10,21-23^ Two of these SRs selected 16 RCTs;^22,23^ one analyzed 23 articles (both clinical trials and prospective studies); and the review conducted to update the IWGDF guidelines included 32 articles.^10^

The SR by Nelson et al.^21^ included 23 studies, identifying seven RCTs that compared the following systemic antibiotics used to treat ulcers of the diabetic foot: imipenem-cilastatin, piperacillin-tazobactam, cefazolin, ceftriaxone, ampicillin-sulbactam, cefoxitin, linezolid, and ticarcillin-clavulanate. The included studies described a range of different types of therapeutic intervention and some included cases with infections of skin and adjacent tissues in their samples. The authors highlighted the variability of outcomes and the small sample sizes of the analyzed studies, causing low statistical power and low quality evidence.^21^

In 2018, Tchero et al.^22^ published an SR including 16 articles that made 10 comparisons between the following systemic antibiotics: piperacillin-tazobactam (followed or not by amoxycillin-clavulanate), moxifloxacin, ertapenem, ampicillin-sulbactam, imipenem-cilastatin, ticarcillin-clavulanate, ceftriaxone (with or without metronidazole), levofloxacin (with metronidazole), and tigecycline. This group of authors emphasized the need for better quality evidence because of the elevated heterogeneity of the inclusion criteria, the sample size, and the time at which outcomes were assessed, concluding that ertapenem achieved better results than tigecycline and worse results when compared to piperacillin-tazobactam for severe infections.^22^

The SR by Pratama et al.,^23^ from 2022, analyzed 16 studies, including nine comparisons between different systemic antibiotics, as follows: ertapenem, piperacillin-tazobactam, ampicillin-sulbactam, moxifloxacin, amoxycillin-clavulanate, imipenem-cilastatin, tigecycline, ceftriaxone, and levofloxacin. They highlighted the heterogeneous nature of these studies and concluded that there was no strong evidence to recommend any specific antibiotic regime.

The SR conducted to update the IWGDF guidelines, published in 2024, considered several different types of intervention for treatment of diabetic foot infections, including use of antibiotics, duration of treatment, and adjuvant therapies.^10^ The authors selected 32 articles and emphasized the need for studies presenting higher quality evidence. This SR concluded that there were no differences in clinical outcomes for the majority of the antibiotics studied, except for tigecycline, which did not meet the criteria for non-inferiority to ertapenem (with or without vancomycin).^10,14^

In summary, two SRs concluded that there was no clear evidence in favor of one specific antibiotics;^21,23^ one review^22^ identified two RCTs that found significant differences, one in a comparison between ertapenem and tigecycline^14^ and the other in a comparison of ertapenem against piperacillin-tazobactam in a subset of patients with severe infections;^20^ and the SR conducted for the IWGDF suggested that results were similar for the antibiotics compared, with the exception of tigecycline, highlighting the need for higher quality studies.^10^

### Meta-analyses

This integrative review also included two meta-analyses published on the Cochrane database that studied antibiotic therapy for foot wounds in people with DM, one from 2013^24^ and the other from 2015.^25^

The meta-analysis by Gurusamy et al.^24^ studied antibiotics used to treat infections caused by methicillin-resistant *Staphylococcus aureus* (MRSA) in several types of non-surgical wounds. After the selection stages, three RCTs were included in the analysis, totaling 47 MRSA infected ulcers in people with DM. It was not possible to conduct a meta-analysis because of different outcomes and low evidence quality. The antibiotics tested were vancomycin (or semi-synthetic penicillins), daptomycin, ertapenem, piperacillin-tazobactam (followed or not by amoxycillin-clavulanate), and moxifloxacin. The authors concluded that there was no evidence to recommend a specific antibiotic for MRSA infections in these case.^24^

In 2015, a meta-analysis by Selva Olid et al.^25^ selected 20 clinical trials (24 articles) totaling 3,791 cases of foot infections in people with DM. The authors pointed out that only five of these studies attained the sample size originally calculated. According to the meta-analysis,^25^ 18 studies reported the chemical-pharmaceutical industry as the source of research funding, one stated that no pharmaceutical firm funding had been received,^18^ and another did not mention this information.^25^ The antibiotics classes compared were subdivided into antipseudomonal penicillins, wide spectrum penicillins, cephalosporins, carbapenems, fluoroquinolones, and others (daptomycin, vancomycin, linezolid, clindamycin, and tigecycline). The authors concluded that the results did not identify a specific antibiotic regime that achieved better results for treatment of these infections, except for tigecycline, which was significantly less effective than ertapenem (combined or not with vancomycin), and highlighted the many limitations of the studies and the low quality of their evidence.^25^

**Figure 4:**
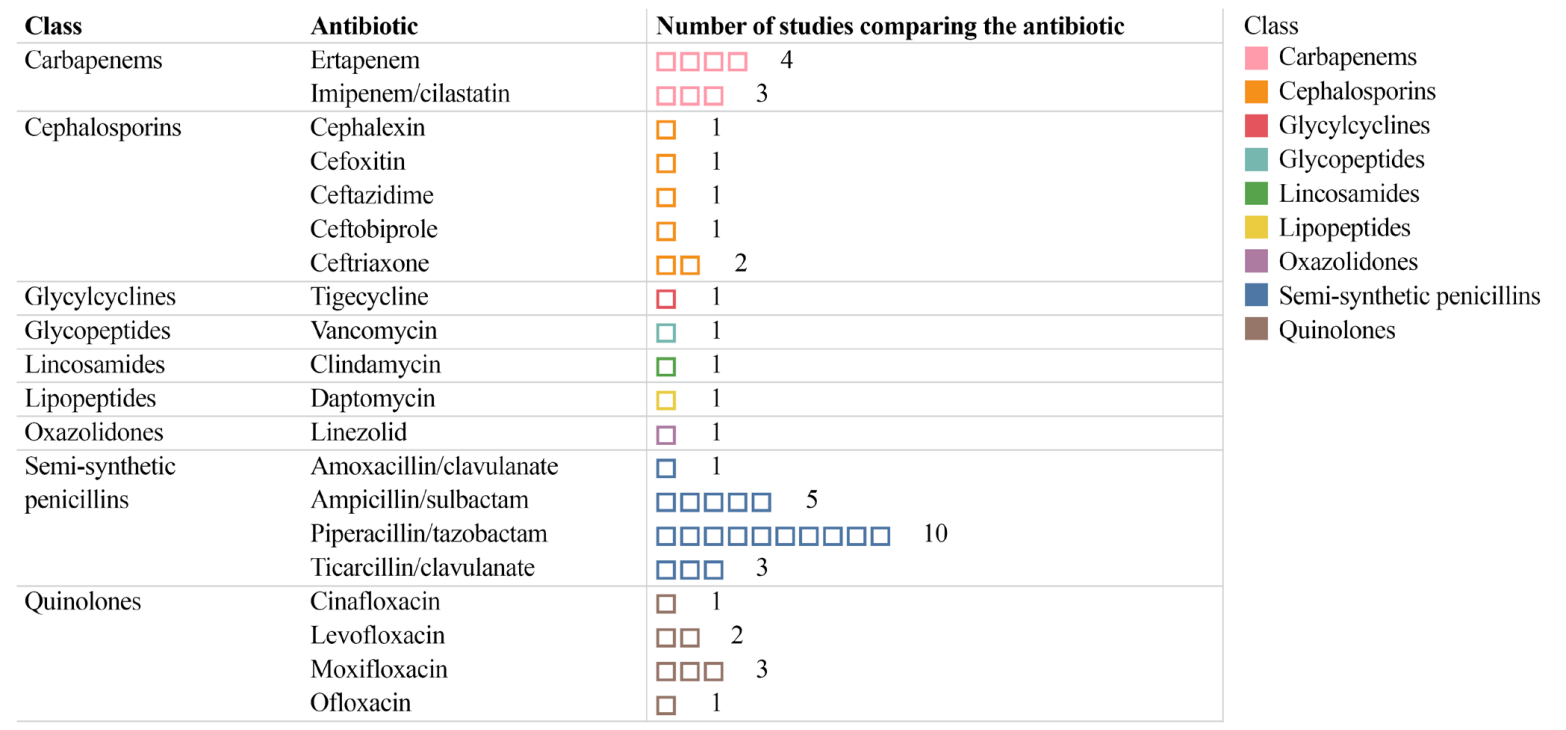
Graph illustrating the frequency of the main systemic antibiotics compared in the included systematic reviews, meta-analyses and the nine randomized clinical trials*.*

The frequencies of each comparison of systemic antibiotics found in the literature, in addition to their classes,^26^ considering the RCTs, SRs, and meta-analyses included in this integrative review are illustrated in Figure 4. Piperacillin-tazobactam was the antibiotic therapy most frequently compared in the RCTs and SRs that studied systemic antibiotics for treatment of DM-related lower limb wound infections.^25^Table 1 summarizes the main conclusions of the RCTs, SRs, and meta-analyses included in this integrative review.

## DISCUSSION

This integrative literature review of the efficacy of systemic antibiotics to treat diabetic foot infections found that, except for tigecycline, which was inferior to ertapenem (with or without vancomycin) in one of the RCTs,^14^ there were no significant differences in the efficacy of the majority of antibiotics when they were compared. Analysis of a subset of patients with severe infections in another RCT^20^ suggested that piperacillin-tazobactam achieved better clinical results than ertapenem.

It is important to point out that the RCTs that compared systemic antibiotics for treatment of the diabetic foot had a variety of different sample sizes and differing outcome definitions, which could interfere with comparison of their results. There were also diverse classifications of the clinical severity of the ulcers and differing follow-up periods. The classification most often used was the University of Texas system,^27^ used in three RCTs, two studies did not mention any ulcer classification system, and none of the RCTs used the WIfI classification (Wound, Ischemia, and foot Infection), published by the Society for Vascular Surgery in 2014.^5^ Underscoring the methodological differences between the studies, the four SRs and two meta-analyses analyzed in this article all emphasized the need for better quality evidence on antibiotic treatment of infections of DM-related injuries.

Selection of antibiotics to treat diabetic foot infections remains a challenge for the literature and for day-to-day clinical practice. Multiple factors contribute to the therapeutic complexity involved. The first is the microbiology of these infections. A systematic review of studies undertaken in different countries revealed a diverse microbiological profile of DM-related foot infections, identifying a predominance of Gram-positive germs in countries with high incomes and of Gram-negative microbes in lower income countries.^28^ Studies in Brazil diverge on the microbiological profile of foot infections among people with DM, describing both Gram-positive^29,30^ and Gram-negative^31,32^ predominance. The majority of authors of the studies reviewed here found gram-positive bacteria predominated and the only RCT which found Gram-negative predominance observed clinical success rates that were in general below 50%. The literature describes increasing rates of antibiotic resistance worldwide and Gram-negative strains are among the species that most concern health organizations because of their high prevalence of resistance to many classes of antibiotics.^33^

Another point to be considered, and one that contributes to the difficulty of selecting the ideal antibiotic therapy in these cases, is the multitude of different classifications of DM-related lower limb ulcers, with depth, severity of infection, and presence of ischemia converging to make treatment more complex.^34^ The pathophysiology of the different stages of diabetic ulcers can include neuropathy, infection, and ischemia, in combination or in isolation.^5,27,34^ The Wagner classification only covers the characteristics of the wound, whereas the PEDIS system combines information on perfusion, extension/depth of the wound, severity of the infection, and neuropathy.^34^ The majority of the RCTs included were conducted before the publication of the WIfI Classification, which includes information on wound depth, degrees of ischemia, and severity of infection and is intended to make analysis more uniform, determine risk of amputation, and contribute to identifying situations in which revascularization will be beneficial.^5^ Considering that the RCTs included did not use a single classification, it is difficult to compare the clinical results of different antibiotics used to treat wounds that may differ in terms of pathophysiology, clinical severity, and microbiology. There are also certain general factors that make selection of antibiotics more difficult, such as local availability, route of administration, cost, and the lack of studies of other classes of antibiotics.

The objective of this integrative review was to collect and summarize the literature on the results of systemic antibiotics for treatment of diabetic foot infections. After analysis of the RCTs, SRs, and meta-analyses included, it is clear that it is difficult to identify a specific antibiotic regime that should be chosen to treat these infections. However, certain aspects can be highlighted, such as the greater number of studies using beta lactam antibiotics combined with beta-lactamase inhibitors, such as piperacillin-tazobactam, ampicillin-sulbactam, and ticarcillin-clavulanate, with good results. Carbapenems were also used in comparisons between systemic antibiotics, with imipenem-cilastatin obtaining similar results to the others. Ertapenem had a lower rate of favorable clinical results in an analysis of a subset of patients with severe infections in one RCT.^20^ The quinolones ofloxacin and moxifloxacin exhibited similar clinical success rates to the antibiotics they were compared with and the same was true of linezolid and of ceftriaxone combined with metronidazole. Tigecycline did not meet the criteria for non-inferiority when compared with the carbapenem ertapenem, which could be combined with vancomycin in cases with MRSA infection.^14^

This review has some limitations. After a general review of the subject in the literature, it was decided to conduct a systematic search for references on a single database. The PubMed database was chosen because of the large number of periodicals indexed, enabling a larger number of manuscripts to be found and reducing interference from duplicates. A prior consultation using other databases revealed a high number of duplicates with no significant addition of manuscripts. The RCTs employed a diverse range of methodologies and a wide range of sample sizes, different definitions of clinical outcomes, and differing wound classifications. They also differed in terms of the material collected for cultures and the ways they described the microorganisms isolated. Considering these aspects, this review employed a systematic and integrative methodology, providing a detailed synthesis of studies that compared systemic antibiotics for treatment of infections of foot wounds in people with DM, which is an important problem seen in all health care sectors, in Brazil and worldwide.

## CONCLUSIONS

The literature suggests that the clinical results of the different systemic antibiotics studied for treatment of diabetic foot infections are comparable, with a significant difference for tigecycline, which did not meet parameters of non-inferiority to ertapenem. The SRs and meta-analyses also note the need for better quality evidence on comparisons of different antibiotic regimes. Considering the importance of treating foot infections in people with DM and the worldwide increase in antibiotic resistance, it is important to conduct studies that can contribute to guiding treatment approaches.

## Data Availability

Os dados analisados nesta revisão são provenientes de estudos publicados na literatura e estão incluídos na tabela e nos gráficos do artigo.
