# Supplementary material for: Systemic antibiotics for diabetes mellitus‐related foot infections: an integrative literature review
Source: J Vasc Bras. 2026 Mar 23;25:e20250097. doi: 10.1590/1677-5449.202500972 (PMC13021003; doi:10.1590/1677-5449.202500972)
Supplement: Translation

# Translation: Antibióticos sistêmicos nas infecções do pé relacionadas ao diabetes mellitus: uma revisão integrativa da literatura

**DOI:** 10.1590/1677-5449.202500971

**Authors:** Catarina França dos Santos, Vanessa Prado dos Santos, Lucas de Mello Ferreira, Neima Prado dos Santos, André Brito Queiroz, Valter Castelli, Roberto Augusto Caffaro, Carlos Alberto Silveira Alves

**Affiliations:** 1 Universidade Federal da Bahia – UFBA, Salvador, BA, Brasil.; 2 Irmandade Santa Casa de Misericórdia de São Paulo – ISCMSP, São Paulo, SP, Brasil.

**Keywords:** antibióticos, pé diabético, infecção, tratamento, diabetes melito

## Abstract

As evidências acerca da antibioticoterapia nas infecções do pé diabético podem auxiliar na terapêutica. O objetivo deste estudo foi identificar as evidências acerca dos antibióticos sistêmicos no tratamento das infecções do pé relacionadas ao diabetes melito. Foi realizada uma revisão integrativa da literatura, incluindo ensaios clínicos randomizados, revisões sistemáticas e metanálises. As palavras-chave “Diabetic foot” AND “Antibiotics” foram utilizadas como descritores na PubMed, sendo selecionados 15 artigos (nove ensaios clínicos randomizados, quatro revisões sistemáticas e duas metanálises). Sete ensaios clínicos randomizados revelaram resultados clínicos comparáveis para os betalactâmicos com inibidores de betalactamases, carbapenêmicos e fluoroquinolonas. Dois ensaios clínicos randomizados mostraram diferenças significativas na comparação entre ertapenem e tigeciclina e na análise do subgrupo de infecções graves entre piperacilina-tazobactam e ertapenem. A literatura aponta resultados clínicos comparáveis para os antibióticos sistêmicos no tratamento das infecções do pé relacionadas ao diabetes, exceto pela diferença entre ertapenem e tigeciclina, que não atingiu os parâmetros de não inferioridade, destacando a necessidade de evidências de maior qualidade.

## INTRODUÇÃO

O diabetes melito (DM) representa um desafio à saúde pública no mundo todo, apresentando uma crescente prevalência. No ano de 2021, havia 529 milhões de pessoas portadoras de DM, e a estimativa é de que esse número chegue a mais de 1,31 bilhão em 2025^1^. A prevalência de DM, padronizada por idade, aumentou 90,5% entre 1990 e 2021, ascendendo de 3,2% para 6,1%, sendo que, na América do Sul, esse aumento foi de mais de 100%^1^. A maioria dos casos (96,0%) é de DM tipo 2 e, de maneira geral, os homens apresentam maior prevalência, exceto em algumas regiões geográficas, como América Latina Central, Sul da África Subsaariana e Caribe, onde a prevalência é maior entre as mulheres^1^.

A hiperglicemia do DM está relacionada a diversas complicações agudas e crônicas, entre elas as úlceras do pé em pessoas com DM. A prevalência estimada de ulceração no pé relacionada ao DM é de 6,3%, sendo maior entre as pessoas com DM tipo 2, em comparação ao tipo 1 (6,4% *versus* 5,5%)^2^. As lesões ou úlceras do pé diabético podem ter diferentes etiologias, como neuropatia periférica, isquemia por doença vascular ou mesmo a associação entre os dois fatores, resultando nas úlceras neuroisquêmicas, que são as mais frequentes^3^. Estima-se que 19 a 34% das pessoas portadoras de DM irão desenvolver uma úlcera ou ferida nos pés ao longo de suas vidas^3,4^.

Na literatura, existem numerosas classificações para as feridas do pé relacionadas ao DM, que consideram diversos aspectos que denotam gravidade, como tamanho e profundidade da lesão, neuropatia, isquemia e presença de infecção^3^. A presença de isquemia e infecção nessas lesões torna o tratamento mais complexo e aumenta o risco de amputações^3,5^. As infecções nas feridas do pé relacionadas ao DM podem ser classificadas em leves, moderadas e graves, de acordo com as características locais e sistêmicas da doença^6^. Nas lesões infectadas, além dos cuidados com a ferida, frequentemente é necessária a introdução de antibioticoterapia empírica, até que haja o resultado de culturas e antibiogramas.

Nas infecções moderadas e graves, as diretrizes do International Working Group on the Diabetic Foot (IWGDF) recomendam a coleta de culturas, preferencialmente de tecido, para orientar a terapêutica antimicrobiana direcionada ao patógeno causador da infecção^7^. Para a seleção da antibioticoterapia nas feridas do pé relacionadas ao DM, é importante observar diversos aspectos, tais como parâmetros clínicos do doente, a gravidade da ferida, o uso prévio de antibióticos, os microrganismos mais implicados e, ainda, conhecer as evidências da literatura a respeito da eficácia dos diferentes antimicrobianos nesse tipo de infecção^7,8^.

O objetivo deste estudo foi identificar, por meio de uma revisão integrativa da literatura, quais são as evidências científicas a respeito da eficácia dos antimicrobianos sistêmicos no tratamento das infecções do pé relacionadas ao DM. Por meio desta revisão, pretende-se verificar se as evidências da literatura apontam para a seleção de antibióticos específicos no tratamento das infecções das lesões do pé em pessoas portadoras de DM.

## MÉTODOS

O estudo contou com uma metodologia de revisão integrativa da literatura, que adotou os critérios estabelecidos pelos *Preferred Reporting Items for Systematic Reviews and Meta-Analyses* para a busca sistematizada das referências^9^.

### Estratégia de busca

Foram selecionadas, entre os Descritores em Ciências da Saúde (DeCS) e o *Medical Subject Headings* (MeSH), as palavras-chave “Diabetic foot” AND “Antibiotics”. A base de dados utilizada para a busca de artigos foi a PubMed, realizando a busca por referências por meio de três etapas, utilizando os mesmos descritores. Na primeira etapa, foi aplicado o filtro para ensaios clínicos e ensaios clínicos randomizados (ECRs). Na segunda etapa, aplicou-se o filtro para revisões sistemáticas (RSs) e, na terceira etapa, o filtro para metanálises. A terceira etapa da busca por artigos foi concluída em 8 de janeiro de 2025.

### Critérios de elegibilidade

Foram considerados elegíveis ensaios clínicos (randomizados ou não), RSs e metanálises que trouxessem a comparação entre diferentes antibióticos sistêmicos para o tratamento das lesões infectadas do pé relacionadas ao DM (pé diabético). Não foram considerados elegíveis os artigos que compararam antibióticos tópicos, que estudaram a duração da antibioticoterapia ou a associação de outros tipos de tratamento para as lesões do pé em pessoas portadoras de DM.

### Seleção dos artigos

A partir dos descritores, antes da aplicação dos filtros nas três etapas da revisão, a busca por artigos na base de dados PubMed resultou em 2.200 resultados. Após a aplicação dos filtros, foram reunidos 212 títulos que, após aplicados os critérios de seleção, resultaram em 15 publicações que integram esta revisão. Todos os artigos encontrados na busca tiveram seus títulos lidos, considerando o objetivo da pesquisa e os critérios de seleção estabelecidos. Os resumos elegíveis foram lidos considerando os mesmos critérios.

Seguidas as etapas de busca e aplicados os critérios de elegibilidade, foram selecionados para a análise nesta revisão integrativa nove ECRs, quatro RSs da literatura e duas metanálises. Uma das RSs selecionadas^10^ continha a revisão da literatura associada às diretrizes do IWGDF, em conjunto com a Infectious Diseases Society of America^7^. O fluxograma de inclusão das publicações encontra-se detalhado na Figura 1. A busca e seleção de artigos para esta revisão integrativa foram realizadas por dois pesquisadores de maneira independente. As discordâncias foram sanadas por meio da discussão entre os autores. Foi elaborada uma planilha no Microsoft Excel para extração e análise dos dados dos estudos incluídos.

**Figura 1:** Fluxograma do processo de seleção dos artigos incluídos na revisão integrativa da literatura^11^. EC = ensaio clínico; ECR = ensaio clínico randomizado; RS = revisão sistemática.

### Metodologia de análise dos artigos selecionados

Os 15 artigos selecionados foram analisados de acordo com uma planilha do Microsoft Excel para extração de dados, com informações detalhadas a respeito dos estudos incluídos, suas principais características e resultados da comparação entre antibióticos sistêmicos. Foram extraídas informações sobre autores, ano da publicação, periódico, tamanho da amostra, objetivos, metodologia, antibióticos sistêmicos comparados, eficácia dos antimicrobianos/sucesso clínico e conclusões dos autores. As porcentagens de sucesso clínico foram aproximadas matematicamente para a confecção dos gráficos. Os artigos incluídos foram analisados de maneira descritiva e tiveram seus dados visualizados por meio do *software* Tableau®.

## RESULTADOS

### Ensaios clínicos randomizados

Foram incluídos nove ECRs que comparavam a eficácia de diferentes antibióticos sistêmicos para o tratamento das infecções nas lesões do pé em pessoas portadoras de DM (Tabela 1)^12-20^. A amostra analisada nos ECRs variou entre 62 e 944 casos (Figura 2). A forma de classificar as lesões tratadas com antibioticoterapia diferiu entre os nove ECRs (Tabela 2). Dois ECRs mencionaram a escala de Wagner^12,18^, três relataram as úlceras como lesões mais frequentes^13,15,16^, dois descreveram apenas a classificação da Universidade do Texas^17,20^, um apenas a classificação PEDIS (abreviação de *perfusion, extent/size, depth/tissue loss, infection and sensation*)^14^ e um utilizou duas classificações (PEDIS e Universidade do Texas)^19^.

**Tabela 1:** Síntese dos objetivos, antibióticos sistêmicos comparados e conclusões dos ensaios clínicos randomizados, revisões sistemáticas e metanálises incluídos.

**Autores**	**Síntese dos objetivos**	**Amostra analisada/número de estudos incluídos**	**Antibióticos comparados**	**Resumo das conclusões**
**Ensaios clínicos randomizados**				
Clay et al.^12^	Comparar metronidazol + ceftriaxona *versus* ticarcilina/clavulanato	70	Metronidazol + ceftriaxona *versus* ticarcilina/clavulanato	Metronidazol + ceftriaxona foi tão efetivo quanto ticarcilina/clavulanato
Grayson et al.^13^	Comparar imipenem/cilastatina *versus* ampicilina/sulbactam	96^a^	Imipenem/cilastatina *versus* ampicilina/sulbactam	Eficácia similar de ampicilina/sulbactam e imipenem/cilastatina
Lauf et al.^14^	Comparar tigeciclina *versus* ertapenem (com ou sem vancomicina)	944	Tigeciclina *versus* ertapenem (com ou sem vancomicina)	Tigeciclina não atendeu aos critérios de não inferioridade quando comparada ao ertapenem (± vancomicina)
Lipsky et al.^15^	Comparar floxacina (seguida de ofloxacina oral) *versus* ampicilina/sulbactam (seguida de amoxicilina clavulanato oral)	88	Ofloxacina (seguida de ofloxacina oral) *versus* ampicilina/sulbactam (seguida de amoxicilina/ clavulanato oral)	Ambos os regimes foram efetivos, resultando em cura ou melhora na maioria dos casos
Lipsky et al.^16^	Comparar linezolida *versus* aminopenicilina/inibidor de betalacmatase	311	Linezolida *versus* ampicilina/sulbactam e/ou amoxicilina/clavulanato (possível associar vancomicina ou aztreonam)	Linezolida foi tão eficaz quanto aminopenicilina/inibidor de betalactamase
Lipsky et al.^17^	Comparar ertapenem *versus* piperacilina/tazobactam	445	Ertapenem *versus* piperacilina/tazobactam	Ertapenem foi tão efetivo quanto piperacilina/tazobactam
Saltoglu et al.^18^	Comparar piperacilina/tazobactam *versus* imipenem/cilastatina	62	Piperacilina/tazobactam *versus* imipenem/cilastatina	Apesar da melhor resposta clínica da piperacilina/tazobactam, a diferença não foi estatisticamente significante
Schaper et al.^19^	Comparar moxifloxacina *versus* betalactâmico/inibidor de betalactamase	206	Moxifloxacina (seguida de moxifloxacina oral) *versus* piperacilina/tazobactam (seguida de amoxicilina/clavulanato)	Moxifloxacina foi eficaz e similar à piperacilina/tazobactam.
Xu et al.^20^	Comparar ertapenem *versus* piperacilina/tazobactam	443	Ertapenem *versus* piperacilina/tazobactam	Ertapenem não foi inferior à piperacilina/tazobactam no geral, mas teve taxa significativamente menor de resolução clínica nas infecções graves
**Revisões sistemáticas**				
Nelson et al.^21^	Clínica e custo-efetividade dos agentes antimicrobianos para as úlceras do pé diabético	23	Imipenem-cilastatina, piperacilina-tazobactam, cefazolina, ceftriaxona, ampicilina-sulbactam, cefoxitina, linezolida, ticarcilina-clavulanato	As evidências são fracas para recomendar um agente antimicrobiano específico
Tchero et al.^22^	Eficácia dos antibióticos tópicos e sistêmicos nas infecções do pé diabético	16	Piperacilina-tazobactam (seguido ou não por amoxicilina-clavulanato), moxifloxacina, ertapenem, ampicilina-sulbactam, imipenem-cilastatina, ticarcilina-clavulanato, ceftriaxone (com metronidazol), tigeciclina	Resultados melhores do ertapenem quando comparado à tigeciclina, mas inferior à piperacilina/tazobactam no tratamento das infecções graves
Pratama et al.^23^	Eficácia dos vários regimes de antibióticos em pacientes com infecção de úlceras do pé diabético	16	Ertapenem, piperacilina-tazobactam, ampicilina-sulbactam, moxifloxacina, amoxicilina-clavulanato, imipenem-cilastatina, tigeciclina, ertapenem, ceftriaxona, levofloxacina	Não há evidências fortes que recomendem um antibiótico específico com maior eficácia
Peters et al.^10^	Intervenções terapêuticas para as diretrizes do IWGDF^b^ sobre o diagnóstico e tratamento das infecções do pé diabético	32^c^	Betalactâmicos/inibidores betalactamases; imipenem-cilastatina; cefoxitina; ofloxacina; linezolida; ertapenem; tigeciclina; moxifloxacina	Não houve diferença nos resultados entre antibióticos comparados, exceto no estudo que encontrou inferioridade da tigeciclina em relação ao ertapenem (com ou sem vancomicina).
**Metanálises**				
fGurusamy et al.^24^	Comparar os antibióticos no tratamento de feridas não cirúrgicas com infecção ou colonização por MRSA^d^	3	Daptomicina, vancomicina, penicilina semissintética, ertapenem, moxifloxacina, piperacilina-tazobactam seguida ou não por amoxicilina-clavulanato	Ausência de evidências para recomendar um antibiótico específico para infecções de feridas não cirúrgicas por MRSA^c^
Selva Olid et al.^25^	Comparar antibióticos sistêmicos no tratamento de infecções do pé diabético com outros antibióticos sistêmicos, tópicos ou placebo	20^e^	piperacilina-tazobactam; ticarcilina-clavulanato; ampicilina-sulbactam; aztreonam; imipenem-cilastatina; cefoxitina; ceftobiprole; ceftazidima; vancomicina; ceftriaxona + metronidazol; ertapenem; moxifloxacina; cinafloxacina; amoxicilina-clavulanato; levofloxacina; ofloxacina; daptomicina; linezolida; clindamicina; cefalexina; tigeciclina	Não foi identificado um antibiótico específico para melhores resultados na resolução da infecção; um estudo apontou que ertapenem (± vancomicina) foi mais efetivo que tigeciclina

MRSA = *Methicillin-resistant Staphylococcus aureus*; IWGDF = International Working Group on the Diabetic Foot.

a 96 eventos/92 pacientes.

b IWGDF.

c 26 estudos na análise para a revisão a respeito da antibioticoterapia sistêmica.

d MRSA.

e 20 estudos/24 artigos.

f De acordo com os autores, não foi possível realizar a metanálise.

**Figura 2:** Representação gráfica da amostra para cada antibiótico comparado nos nove ensaios clínicos randomizados.

**Tabela 2:** Antibióticos, classificações das lesões e principais resultados microbiológicos dos ensaios clínicos randomizados incluídos nesta revisão integrativa.

**Autores**	**Sucesso clínico dos antimicrobianos comparados (%)**	**Amostra analisada**	**Classificações das lesões dos membros inferiores**	**Tipo de patógeno predominante**	**Principais espécies/gêneros de microrganismos isolados (n)**
**Ensaios clínicos randomizados**					
Clay et al.^12^	Ceftriaxona + metronidazol (72%) *versus* ticarcilina-clavulanato (76%)	70	Wagner 1-3 (71% dos pacientes apresentavam celulite do pé)	Gram-positivo	*Staphylococcus aureus* (20); *Streptococcus* grupo B (9); *Enterococcus faecalis* (6)
Grayson et al.^13^	Imipenem-cilastatina (85%) *versus* ampicilina-sulbactam (81%)	96^a^	Úlceras (88 casos)	Gram-positivo	*Staphylococcus aureus* (54); *Enterococcci* (35); *Streptococci* (28)
Lauf et al.^14^	Tigeciclina (77,5%) *versus* ertapenem (82,5%) (com ou sem vancomicina)	944	PEDIS graus de infecção 2-4 (90% PEDIS 2 ou 3)	Gram-positivo	*Staphylococcus aureus* (323); *Enterococcus faecalis* (134); *S. agalactiae* (88)
Lipsky et al.^15^	Ofloxacina (85%) (seguida de ofloxacina oral) *versus* ampicilina-sulbactam (83%) (seguida de amoxacilina-clavulanato oral)	88	Úlceras infectadas (48 casos), celulites (26 casos)	Gram-positivo	*Staphylococcus aureus* (41); *Streptococcus sp*. (24); *Staphylococcus* coagulase-negativo (16)
Lipsky et al.^16^	Linezolida (81%) *versus* aminopenicilina-inibidor de betalacmatase (71%)	311	Úlceras infectadas (283 casos), celulites (161 casos)	Gram-positivo	*Staphylococcus aureus* (131); *Staphylococcus* coagulase-negativo (54); *Enterococcus sp.* (51)
Lipsky et al.^17^	Ertapenem (94%) *versus* piperacilina-tazobactam (92%)	445	Universidade do Texas (mais comum, moderado grau I, estágio-B: 354 casos)	Gram-positivo	*Staphylococcus aureus* (147), *Peptostreptococcus magnus* (65), *Streptococcus agalactiae* (46)
Saltoglu et al.^18^	Piperacilina-tazobactam (46.7%) *versus* imipenem-cilastatina (28,1%)	62	Wagner 2-4 (34 casos classe 3; 19 casos classe 4)	Gram-negativo	*Staphylococcus* coagulase-negativo (15); *Pseudomonas aeruginosa* (13); *Enterococcus sp*. (10)
Schaper et al.^19^	Moxifloxacina (76,4%) *versus* piperacilina-tazobactam (78,1%)	206	PEDIS graus 2-4; Universidade do Texas (Grau II-isquêmica: 88 casos e Grau II-infectada: 30 casos)	Gram-positivo	*Staphylococcus aureus* (133), *Enterococcus faecalis* (59), *Escherichia coli* (21)
Xu et al.^20^	Ertapenem (93,6%) *versus* piperacilina-tazobactam (97,3%)	443	Universidade do Texas (Severa-grau 2/ estágio B:131 casos; Moderada-grau 1/estágio B: 106 casos)	Gram-positivo	*Staphylococcus aureus* (126); *Enterococcus faecalis* (72); *Escherichia coli* (51)

a 96 eventos/92 pacientes.

PEDIS = *perfusion, extent/size, depth/tissue loss, infection and sensation*.

Os ECRs diferiram na forma de avaliar os resultados da antibioticoterapia, descrevendo a melhora dos sintomas, cura clínica ou resposta clínica favorável, utilizando critérios distintos para a definição do desfecho. As classes de antibióticos mais comparadas foram os betalactâmicos associados aos inibidores de betalactamase (oito estudos), seguidos dos carbapenêmicos (cinco estudos), fluoroquinolonas (dois estudos) e, em um estudo cada, as cefalosporinas, oxazolidinonas e glicilciclina. No ECR que comparou linezolida *versus* aminopenicilina com inibidor de betalactamase, poderia ser adicionada vancomicina ou aztreonam, a critério da equipe^16^. Considerando os nove ECRs incluídos, as taxas de eficácia, sucesso clínico ou resultado favorável variaram entre 28,1 e 97,3% para os diferentes antibióticos comparados (Tabela 2, Figura 3).

**Figura 3:** Representação gráfica da porcentagem dos resultados do tratamento (sucesso clínico) para os antibióticos comparados nos nove ensaios clínicos randomizados.

De maneira geral, oito dos nove estudos não apresentaram diferenças significativas quanto ao sucesso clínico entre os antimicrobianos comparados. O ECR que obteve diferença significativa encontrou que a tigeciclina não atendeu aos critérios de não inferioridade quando comparada ao ertapenem, que poderia ser associado à vancomicina^14^. Considerando o grupo analisado que recebeu tigeciclina (408 casos), 316 (77,5%) foram considerados curados, o mesmo ocorrendo para 334 (82,5%) dos 405 casos tratados com ertapenem ± vancomicina^14^. A vancomicina foi administrada como tratamento adjuvante ao ertapenem em 15,6% dos casos.^14^ Este ECR^14^ foi o que incluiu o maior número de pacientes, contando com a maior amostra entre os estudos incluídos (Figura 2).

A comparação entre ertapenem *versus* piperacilina-tazobactam no ECR^20^, que incluiu 443 infecções moderadas a graves, não mostrou diferença significativa na resposta clínica da amostra global (93,6% *versus* 97,3%), mas encontrou uma taxa significativamente menor de resposta favorável ao ertapenem na análise do subgrupo de doentes (273 casos) com infecções graves (91,5% *versus* 97,2%; p = 0,04).^20^ Quanto à microbiologia das lesões, em oito ECRs houve predomínio de microrganismos Gram-positivos, notadamente o *Staphylococcus aureus* (Tabela 2). No único ECR^18^ incluído em que houve predomínio de bactérias Gram-negativas, os resultados clínicos mostraram menores taxas de sucesso que os demais estudos (Figura 3).

### Revisões sistemáticas

As quatro RSs incluídas nesta análise reuniram estudos que continham agentes antimicrobianos sistêmicos e tópicos^10,21-23^. Duas das RSs selecionaram 16 ECRs^22,23^; uma reuniu 23 artigos (tanto ensaios clínicos como estudos prospectivos); e a revisão para atualização do IWGDF considerou 32 artigos^10^.

A RS de Nelson et al.^21^ incluiu 23 estudos, identificando sete ECRs que compararam os seguintes antibióticos sistêmicos utilizados para o tratamento das úlceras do pé diabético: imipenem-cilastatina, piperacilina-tazobactam, cefazolina, ceftriaxone, ampicilina-sulbactam, cefoxitina, linezolida e ticarcilina-clavulanato. Os estudos incluídos apresentavam diversos tipos de intervenção terapêutica, e alguns deles consideraram na amostra casos com infecções de pele e tecidos adjacentes. Os autores destacaram a variabilidade dos desfechos e o tamanho reduzido das amostras dos estudos, ocasionando baixo poder estatístico e reduzindo a qualidade das evidências^21^.

Em 2018, Tchero et al.^22^ publicaram uma RS incluindo 16 artigos que traziam 10 comparações entre antibióticos sistêmicos, sendo estudados: piperacilina-tazobactam (seguido ou não por amoxicilina-clavulanato), moxifloxacina, ertapenem, ampicilina-sulbactam, imipenem-cilastatina, ticarcilina-clavulanato, ceftriaxone (com ou sem metronidazol), levofloxacina (com metronidazol) e tigeciclina. O grupo de autores aponta a necessidade de evidências de melhor qualidade, devido à elevada heterogeneidade nos critérios de inclusão, no tamanho da amostra e no tempo para avaliação do desfecho, concluindo melhores resultados do ertapenem em relação à tigeciclina e piores resultados quando comparado à piperacilina-tazobactam em infecções graves^22^.

A RS de Pratama et al.^23^, de 2022, reuniu 16 estudos, entre eles nove comparações entre antibióticos sistêmicos, sendo: ertapenem, piperacilina-tazobactam, ampicilina-sulbactam, moxifloxacina, amoxicilina-clavulanato, imipenem-cilastatina, tigeciclina, ceftriaxona e levofloxacina, destacando a heterogeneidade dos estudos e concluindo pela ausência de evidências fortes que recomendem um esquema antibiótico específico.

A RS realizada para a atualização das diretrizes do IWGDF, publicada em 2024, considerou diferentes tipos de intervenção para o tratamento das infecções do pé relacionadas ao DM, incluindo o uso de antimicrobianos, seu tempo de duração e as terapias adjuvantes^10^. Os autores selecionaram 32 artigos e destacaram a necessidade de estudos que tragam evidências de maior qualidade. A RS concluiu que não houve diferença nos desfechos clínicos para a maioria dos antimicrobianos estudados, exceto para a tigeciclina, que não alcançou os critérios de não inferioridade quando comparada ao ertapenem (*versus* com ou sem vancomicina)^10,14^.

Em síntese, duas RSs concluíram pela ausência de evidências claras em favor de um antimicrobiano específico^21,23^; uma revisão^22^ apontou dois ECRs que encontraram diferenças significativas, um na comparação entre ertapenem e tigeciclina^14^ e outro entre ertapenem e piperacilina-tazobactam no subgrupo de doentes com infecções graves^20^; e a RS para o IWGDF sugeriu resultados semelhantes para os antibióticos comparados, com exceção da tigeciclina, destacando a necessidade de estudos de maior qualidade^10^.

### Metanálises

Foram incluídas nesta revisão integrativa duas metanálises publicadas na Cochrane que estudaram antibioticoterapia nas lesões do pé em pessoas com DM, uma no ano de 2013^24^ e outra em 2015^25^.

A metanálise de Gurusamy et al.^24^ estudou os antimicrobianos utilizados para o tratamento das infecções causadas por *Staphylococcus aureus* resistente à meticilina (MRSA, do inglês *Methicillin-resistant Staphylococcus aureus*) nos diversos tipos de feridas não cirúrgicas. Após as etapas de seleção, foram incluídos na análise três ECRs, reunindo 47 úlceras infectadas por MRSA em pessoas com DM, não sendo possível realizar a metanálise devido aos diferentes desfechos e à baixa qualidade da evidência. Os antibióticos testados foram: vancomicina (ou penicilinas semissintéticas), daptomicina, ertapenem, piperacilina-tazobactam (seguida ou não de amoxicilina-clavulanato) e moxifloxacina. Os autores concluíram que não havia evidência que levasse à recomendação de um antibiótico específico para as infecções por MRSA nesses casos^24^.

Em 2015, a metanálise de Selva Olid et al.^25^ reuniu 20 ensaios clínicos (24 artigos) que somaram 3.791 casos de infecções do pé em pessoas com DM, destacando que apenas cinco estudos atingiram o número inicialmente calculado para a amostra. De acordo com a metanálise^25^, 18 estudos indicaram o setor químico-farmacêutico como fonte de financiamento da pesquisa, um declarou que não houve financiamento por empresa farmacêutica^18^ e outro não trouxe a informação^25^. As classes de antimicrobianos comparadas foram subdivididas em penicilinas antipseudomonas, penicilinas de amplo espectro, cefalosporinas, carbapenêmicos, fluoroquinolonas e outros (daptomicina, vancomicina, linezolida, clindamicina e tigeciclina). Os autores concluíram que os resultados não demonstraram um esquema antimicrobiano específico que produza melhores resultados no tratamento dessas infecções, exceto pela tigeciclina, que foi significativamente menos efetiva que o ertapenem (associado ou não à vancomicina), enfatizando as diversas limitações dos estudos e a baixa qualidade da evidência^25^.

As frequências dos principais antimicrobianos sistêmicos comparados na literatura, além de suas respectivas classes^26^, considerando os ECRs, RSs e metanálises incluídos nesta revisão integrativa, estão representadas na Figura 4. A piperacilina-tazobactam foi o antimicrobiano mais frequentemente comparado nos ECRs e RSs que estudaram antibióticos sistêmicos para o tratamento das infecções das lesões dos membros inferiores relacionadas ao DM^25^. A Tabela 1 sintetiza as principais conclusões dos ECRs, RSs e metanálises incluídos nesta revisão integrativa.

**Figura 4:** Representação gráfica da frequência dos principais antibióticos sistêmicos comparados nas revisões sistemáticas, metanálises e nos nove ensaios clínicos randomizados incluídos.

## DISCUSSÃO

Esta revisão integrativa da literatura a respeito da eficácia dos antibióticos sistêmicos para o tratamento das infecções do pé relacionadas ao DM encontrou que, exceto pela tigeciclina, que foi inferior ao ertapenem (com ou sem vancomicina) em um dos ECRs^14^, a maioria dos antimicrobianos não apresentou diferença significativa em sua eficácia clínica quando comparados. A análise de um subgrupo de doentes com infecções graves em outro ECR^20^ sugeriu que a piperacilina-tazobactam obteve melhores resultados clínicos que o ertapenem.

É importante destacar que os ECRs que comparam o uso de antibióticos sistêmicos no tratamento do pé diabético contaram com tamanhos variados de amostra e diferentes definições de desfecho, o que pode dificultar a comparação entre os resultados. Houve ainda diversidade de classificações de gravidade clínica da lesão, bem como do período de seguimento dos casos. A classificação mais utilizada foi a da Universidade do Texas^27^, em três ECRs, sendo que dois estudos não mencionaram uma classificação das lesões e nenhum dos ECRs utilizou a classificação WIfI (abreviação do inglês, *Wound, Ischemia, and foot Infection*), publicada pela *S*ociety for Vascular Surgery, em 2014^5^. Reforçando as diferenças metodológicas entre os estudos, as quatro RSs e as duas metanálises incluídas neste trabalho destacam a necessidade de evidências de melhor qualidade sobre o tratamento antimicrobiano das infecções das lesões relacionadas ao DM.

A seleção de antimicrobianos para o tratamento das infecções do pé relacionadas ao DM permanece um desafio para a literatura e para a prática clínica cotidiana. Múltiplos fatores contribuem para essa complexidade terapêutica. O primeiro deles é a microbiologia dessas infecções. Uma revisão sistemática com estudos realizados em diferentes países revelou que o perfil microbiológico das lesões do pé relacionadas ao DM é diverso, apontando predominância de germes Gram-positivos nos países de renda elevada e de Gram-negativos em países de menor renda^28^. No Brasil, os estudos divergem acerca do perfil microbiológico das infecções do pé em pessoas com DM, descrevendo predominância tanto de Gram-positivos^29,30^ como de Gram-negativos^31,32^. Nesta revisão, a maioria dos autores encontrou predominância de bactérias Gram-positivas, sendo que o único ECR que contou com predomínio de Gram-negativos obteve, de maneira geral, índices de sucesso clínico abaixo de 50%. A literatura aponta crescentes índices de resistência antimicrobiana no mundo, sendo que os isolados Gram-negativos estão entre as principais espécies que preocupam as organizações de saúde, devido à sua elevada prevalência de resistência às diversas classes de antibióticos^33^.

Outro ponto a ser considerado, e que contribui para a dificuldade de selecionar a terapêutica antimicrobiana ideal nesses casos, é a multiplicidade de classificações das lesões de membros inferiores associadas ao DM, com profundidade, gravidade da infecção e presença de isquemia confluindo para tornar o tratamento mais complexo^34^. As lesões dos portadores de DM podem ter, em sua fisiopatologia, neuropatia, infecção e isquemia, associadas ou não, nos diversos estágios^,26,33^. A classificação de Wagner contempla apenas as características da ferida, enquanto o sistema PEDIS reúne informações sobre a perfusão, extensão/profundidade da lesão, gravidade da infecção e neuropatia^34^. A maioria dos ECRs incluídos foi realizada em período anterior à publicação da classificação WIfI, que reúne informações sobre a profundidade da ferida, graus de isquemia e gravidade da infecção, buscando tornar a análise mais homogênea, determinar o risco de amputação e contribuir para identificar as situações com benefício da revascularização^5^. Considerando que os ECRs incluídos não utilizaram uma classificação única, é difícil comparar os resultados clínicos de diferentes antibióticos em lesões que podem ter fisiopatologia, gravidade clínica e microbiologia distintas. Existem ainda aspectos gerais que dificultam a seleção dos antimicrobianos, tais como a disponibilidade local, a via de administração, o custo e a falta de estudos acerca de outras classes de antimicrobianos.

Esta revisão integrativa propôs reunir e sintetizar a literatura a respeito dos resultados dos antimicrobianos sistêmicos no tratamento das infecções do pé relacionadas ao DM. Após a análise dos ECRs, RSs e metanálises incluídos, verificou-se a dificuldade em apontar um esquema antibiótico específico a ser selecionado para o tratamento dessas infecções. No entanto, alguns aspectos podem ser destacados, como a maior frequência de estudos utilizando antibióticos betalactâmicos associados aos inibidores de betalactamases, como a piperacilina-tazobactam, ampicilina-sulbactam e ticarcilina-clavulanato, com bons resultados. Os carbapenêmicos também foram utilizados nas comparações entre antibióticos sistêmicos, sendo que o imipenem-cilastatina obteve resultados semelhantes aos demais. O ertapenem obteve uma menor taxa de resultado clínico favorável na análise de um subgrupo de doentes com infecções graves em um ECR^20^. As quinolonas, ofloxacina e moxifloxacina, apresentaram taxas de sucesso clínico semelhantes aos antibióticos comparados, o mesmo ocorrendo com a linezolida e com o ceftriaxone associado ao metronidazol. A tigeciclina não preencheu os critérios de não inferioridade quando comparada ao carbapenêmico ertapenem, que poderia ser associado à vancomicina nos casos de infecção por MRSA^14^.

A revisão apresentada conta com algumas limitações. Após uma pesquisa geral sobre o tema na literatura, optou-se por realizar a busca sistemática por referências em uma única base de dados. A seleção da base informatizada PubMed ocorreu devido à ampla indexação de periódicos científicos na plataforma, possibilitando a busca por um maior número de manuscritos e reduzindo a interferência de duplicatas. Uma consulta prévia a outras bases de dados revelou um elevado número de duplicatas, sem a adição significativa de manuscritos. Os ECRs contaram com metodologias diversas, com ampla variação do tamanho da amostra, diferentes definições de desfecho clínico e distintas classificações das lesões entre os estudos, bem como variabilidade do material coletado para cultura e da forma de descrição dos microrganismos isolados. Considerando esses aspectos, esta revisão contou com uma metodologia sistematizada e integrativa, fornecendo uma síntese detalhada dos estudos que comparam os antibióticos sistêmicos no tratamento de infecções das lesões do pé em pessoas com DM, um importante problema que atinge todos os setores da saúde, no Brasil e no mundo.

## CONCLUSÃO

A literatura sugere que os resultados clínicos dos diferentes antibióticos sistêmicos estudados para o tratamento das infecções do pé relacionadas ao DM são comparáveis, havendo diferença significativa para a tigeciclina, que não atingiu os parâmetros de não inferioridade quando comparada ao ertapenem. As RSs e metanálises apontam, ainda, a necessidade de evidências de melhor qualidade comparando diferentes esquemas antimicrobianos. Considerando a importância do tratamento das infecções do pé em pessoas com DM e o aumento global nos níveis de resistência antimicrobiana, faz-se importante o desenvolvimento de pesquisas que possam contribuir para direcionar a abordagem terapêutica.

## Data Availability

The data analyzed in this review are sourced from published studies available in the literature and are summarized in the table and graphs within the article.
